# Nanosecond pulsed electric field ablation‐induced modulation of sphingolipid metabolism is associated with *Ly6c2* ^+^ mononuclear phagocyte differentiation in liver cancer

**DOI:** 10.1002/1878-0261.13372

**Published:** 2023-01-21

**Authors:** Jingqi Liu, Chengyu Fang, Xinyan Jin, Guo Tian, Zhongxia Sun, Lijie Hong, Jinhua Pan, Xinhua Chen, Jun Zhao, Hongcui Cao, Tianan Jiang

**Affiliations:** ^1^ Department of Ultrasound Medicine The First Affiliated Hospital, Zhejiang University School of Medicine Hangzhou China; ^2^ State Key Laboratory for Diagnosis and Treatment of Infectious Diseases, Collaborative Innovation Center for Diagnosis and Treatment of Infectious Diseases First Affiliated Hospital, Zhejiang University School of Medicine Hangzhou China; ^3^ Key Laboratory of Pulsed Power Translational Medicine of Zhejiang Province Hangzhou China; ^4^ Department of Hepatobiliary and Pancreatic Surgery The First Affiliated Hospital, Zhejiang University School of Medicine Hangzhou China; ^5^ School of Basic Medicine Huazhong University of Science and Technology Wuhan China; ^6^ Zhejiang University Cancer Center Hangzhou China

**Keywords:** immunometabolism, liver cancer, mononuclear phagocyte, nanosecond pulsed electric field

## Abstract

Preclinical studies have proven that nanosecond pulsed electric field (nsPEF) ablation can be a safe and effective treatment for humans with unresectable liver cancer that are ineligible for thermal ablation. The concomitant activation of antitumor immunity by nsPEF can also potentially prevent tumor recurrence. However, whether nsPEF exhibits similar efficacy in a clinical setting remains to be investigated. A prospective clinical trial (clinicaltrials.gov identifier: NCT04039747) was conducted to evaluate the safety and efficacy of ultrasound (US)‐guided nsPEF ablation in 15 patients with unresectable liver cancer that were ineligible for thermal ablation. We found that nsPEF ablation was safe and produced a 12‐month recurrence‐free survival (RFS) and local RFS of 60% (9/15) and 86.7% (13/15), respectively, in the enrolled patients. Integrative proteomic and metabolomic analysis showed that sphingolipid metabolism was the most significantly enriched pathway in patient sera after nsPEF without recurrence within 8 months. A similar upregulation of sphingolipid metabolism was observed in the intratumoral mononuclear phagocytes (MNPs), rather than other immune and nonimmune cells, of an nsPEF‐treated mouse model. We then demonstrated that lymphocyte antigen 6 complex, locus C2‐positive (*Ly6c2* ^+^) monocytes first differentiated into *Ly6c2* ^+^ monocyte‐derived macrophages with an increase in sphingolipid metabolic activity, and subsequently into *Ly6c2* ^+^ dendritic cells (DCs). *Ly6c2* ^+^ DCs communicated with CD8^+^ T cells and increased the proportions of IFN‐γ^+^ CD8^+^ memory T cells after nsPEF, and this finding was subsequently confirmed by depletion of liver *Ly6c2* ^+^ MNPs. In conclusion, nsPEF was a safe and effective treatment for liver cancer. The alteration of sphingolipid metabolism induced by nsPEF was associated with the differentiation of *Ly6c2* ^+^ MNPs, and subsequently induced the formation of memory CD8^+^ T cells with potent antitumor effect.

## Graphical abstract

Nanosecond pulsed electric field (nsPEF) ablation is a safe and effective treatment for patients with liver cancers not amenable to thermal ablation. Alteration of intratumoral sphingolipid metabolism induced by nsPEF ablation was closely related to the differentiation of *Ly6c2* ^+^ mononuclear phagocytes, and which subsequently induced the formation of memory CD8^+^ T cells with potent antitumor effect.

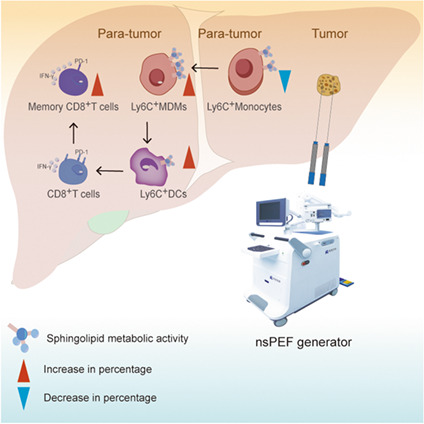

**Abbreviations**
AFP: alpha‐fetoprotein
ALT: alanine transaminase
AST: aspartate aminotransferase
ATCC: America Type Culture Collection
BCA: bicinchonininc acid
BCC: basal cell carcinoma
CEUS: contrast enhanced US
DCE: dynamic contrast enhanced
DCs: dendritic cells
DDA: data‐dependent acquisition
DEGs: differentially expressed genes
DIA: data‐independent acquisition
ESI: electrospray ionization
FC: fold change
FDR: false discovery rate
GO: gene ontology
GSEA: gene set enrichment analysis
HBV: hepatitis B virus
HCC: hepatocellular carcinoma
HMDB: human metabolome database
KEGG: kyoto encyclopedia of genes and genomes
LSECs: liver sinusoidal endothelial cells
*Ly6c2* ^+^
: lymphocyte antigen 6 complex, locus C2‐positive
MDMs: monocyte‐derived macrophages
MNPs: mononuclear phagocytes
MRI: magnetic resonance imaging
MWA: microwave ablation
NK: natural killer
nsPEF: nanosecond pulsed electric field
OPLS‐DA: orthogonal partial least square‐discriminant analysis
OS: overall survival
PCA: principal component analysis
PIVKA‐II: vitamin K antagonist‐II
PPI: protein–protein interaction
QC: quality control
RFA: radiofrequency ablation
RFS: recurrence‐free survival
RT: retention time
scRNA‐seq: single‐cell RNA sequencing
SDMs: significant differential metabolites
SDPs: significant differential proteins
SEM: standard errors of the mean
SPE: solid‐phase extraction
SPF: specified pathogen free
SPSS: statistical package for the social sciences
TAMs: tumor associated macrophages
TME: tumor microenvironment
UMPA: uniform manifold approximation and projection for dimension reduction
UPLC: ultrahigh performance liquid chromatography
US: ultrasound

## 1 Introduction

Liver cancer is one the most common causes of cancer‐related death worldwide. While surgical resection remains the first‐line treatment, most patients are unfit for surgery due to insufficient liver function reserve [1]. This cohort of patients often resort to thermal ablations including radiofrequency ablation (RFA), microwave ablation (MWA), or cryoablation. However, these techniques are restricted from regions adjacent to gallbladder, gastrointestinal tract, hepatic vessels, or poral vessels, because of the presence of delicate structures that are sensitive to thermal damage [2, 3].

Nanosecond pulsed electric field (nsPEF), on the other hand, has recently emerged as a nonthermal ablative treatment for solid tumors. nsPEF utilizes high‐power short electric pulses to disrupt cellular membranes and intracellular structures, e.g. nucleus and mitochondria, and consequently induces the apoptosis and necrosis of tumor cells [4]. It has shown promising efficacy in preclinical and clinical studies on hepatocellular carcinoma (HCC), basal cell carcinoma (BCC), melanoma, and pancreatic cancer [5, 6, 7]. For example, a recent clinical trial has suggested that nsPEF is an effective alternative treatment with less scars and pain than conventional therapy for BCC [5]. Notably, the nonthermal ablation mechanism of nsPEF could spare vital structures from collateral thermal damage as observed in conventional thermal ablation [8]. Previous studies from others and our lab have been reported that nsPEF can eliminate HCC without damaging blood veins, bile ducts, and gallbladder [9, 10]. Furthermore, efficacy of thermal ablation is influenced by dissipation of heat from hepatic or portal veins via blood flow (heat‐sink effect), which results in incomplete ablation of perivascular tumors. Because the main therapeutic principle is not thermal, the efficacy of nsPEF is not limited by the heat‐sink effect [8]. Therefore, it is considered an ideal option for treating tumor nodules close to delicate structures.

We conduct a prospective clinical trial (clinicaltrials.gov. identifier: NCT04309747) to evaluate the safety and efficacy of ultrasound (US)‐guided nsPEF ablation for liver carcinoma who are ineligible for thermal ablation. Our initial results suggested that nsPEF is an effective and safe treatment modality for liver carcinoma who located near gallbladder, hepatic vessels, portal vessels, or gastrointestinal tract.

Importantly, preclinical studies have shown that nsPEF ablation activated antitumor immune response to inhibited tumor recurrence [11, 12]. For example, nsPEF can promote the release of danger‐associated molecular patterns from tumor cells and thus induce CD8^+^ T cell‐mediated antitumor immune response, consequently preventing the tumor growth [13, 14, 15]. However, a recent study has found that the activation of CD8^+^ T cell induced by nsPEF is temporal, 6 days later, activated CD8^+^ T cells reversed into exhausted CD8^+^ T cells due to the release of PD‐L1 from tumor cells [6]. Therefore, it is of great importance to characterize the molecular changes induced by nsPEF ablation starting to advance from a clinical level, and then unveil the specific mechanism of nsPEF ablation modulation of immune response in liver carcinoma. This may help guide the immunotherapies of liver carcinoma patients after nsPEF ablation and discover new liver carcinoma therapeutic targets.

Here, we utilized nontargeted technology to conduct an integrative metabolic and proteomic analysis on serum obtained from naive‐nsPEF ablation liver carcinoma patients without relapse within 8 months. We discovered that the sphingolipid metabolism is the key signaling pathways associated with better outcomes of patients with liver carcinoma after nsPEF ablation. To further investigate nsPEF ablation's role in regulating sphingolipid metabolism of the tumor niche, single‐cell RNA sequencing (scRNA‐seq) analysis was performed to profile immune and metabolic alterations in tumors and adjacent tissues from nsPEF ablation‐naïve orthotopic HCC mice. Furthermore, mass cytometry analysis and selectively depletion of Ly6C^+^ MNPs were performed to validate the scRNA‐seq results. Our results indicated that nsPEF ablation regulated the sphingolipid metabolism of the tumor niche, which is closely related to the differentiation of *Ly6c2* ^+^ MNPs and subsequently promoted the formation of memory CD8^+^ T cells with potent antitumor effect.

## 2 Materials and methods

### 2.1 Patient selection

Fifteen eligible patients 18–75 years of age were consecutively enrolled from June 2020 to August 2020 at the First Affiliated Hospital of Zhejiang University. Criteria for patient inclusion: liver cancer (a) verified by biopsy or noninvasive diagnosis; (b) within 0.5 cm of gallbladder, major portal vein, major hepatic vein, or digestive tract; (c) ineligible for liver transplant, surgical resection, RFA, or MWA; (d) tumor nodules ≤ 5 cm in diameter and no more than 3 nodules in total; (e) absence of extrahepatic metastases by preoperative imaging; (f) Child‐Pugh class A or B; (g) normal coagulation functions: INR ≤ 1.2 × upper limit of normal and platelet count ≥ 5.0 × 10^9^ L^−1^. Criteria for patient exclusion: (a) presence of extrahepatic metastases; (b) invasion to portal or hepatic vein; (c) Child‐Pugh class C; (d) patients with previous refractory ascites, epilepsy, electronic or metal implants near the ablation zone, cardiac arrhythmias, or esophageal variceal hemorrhage within 1 month; (e) participated in other clinical trials within 1 month.

Written informed consent was obtained from all participating patients. The study was conducted in compliance with the principles of the Declaration of Helsinki and was approved by the Ethics Committee of the First Affiliated Hospital, Zhejiang University (approval number: 2019‐333). This study was registered as: NCT04309747 at www.clinicaltrials.gov.

### 2.2 Procedure of nsPEF ablation

Patients underwent general anesthesia with complete muscle paralysis, and the nsPEF procedure was performed using an nsPEF generator (Ruidi Biotechnology Co., Ltd, Hangzhou, China) by certified interventional radiologist with extensive experience in hepatobiliary tumor ablation. Electrodes were placed into or around the liver lesions under US guidance with an optimal intraelectrode distance of 1.0–2.0 cm, delivering pulses of 300 ns in duration and electric voltage of 30 kV. All pulses were given with cardiac synchronization. The number of electrodes placed and pulses delivered were determined according to the diameter of liver lesions. The geometry of electrode placement was designed to optimize the ablation zone based on experience from previous pilot studies. Regular or contrast‐enhanced US was performed immediately after nsPEF to confirm a complete ablation and to monitor any acute complications including bleeding, vessel thrombosis, bile leakage, or intestinal perforation. The detailed parameters of the nsPEF ablation are described in Table 1.

**Table 1:** The detailed parameters of nsPEF ablation.

No. of tumor	No. of electrodes	Electric voltage (kV)	No. of pulse	Electric current (A)	Mean electrode interval (mm)
1	2	30	800	141–145	17.0
2	2	30	1600	144–146	13.0
3	2	30	1600	138–150	14.6
4	2	30	1000	138–141	12.0
5	2	30	2400	144–151	18.8
6	2	30	1000	143–149	12.8
7	2	30	2400	147–152	17.0
8	2	30	800	130–134	18.6
9	2	30	1000	142–145	13.0
10	2	30	2400	145–151	16.2
11	2	30	3200	140–149	18.6
12	2	30	2000	138–144	17.3
13	3	30	2000	138–145	11.3
14	2	30	1800	141–152	15.3
15	2	30	2200	148–155	16.0
16	2	30	2000	145–153	16.0
17	2	30	2000	145–149	11.3

### 2.3 Patient follow‐up

All participating patients underwent diagnostic imaging and routine laboratory tests within 30 days prior to the nsPEF ablation. The diagnostic imaging included US, contrast enhanced US (CEUS), T1‐, T2‐, diffusion‐, and dynamic contrast enhanced (DCE)‐T1‐weighted magnetic resonance imaging (MRI). The laboratory tests consisted of serum transaminase and bilirubin, tumor biomarkers, blood chemistry, coagulation function, and cardiac enzymes. Similar diagnostic imaging and laboratory tests were performed on the patients at 1, 30, 90, and 180 days after the nsPEF ablation, and every 3–6 months thereafter.

Ultrasound and CEUS images were obtained on an ultrasound system (MyLab90 X vision; Esaote, Genoa, Italy) with a 3–13 MHz convex probe. Patients were intravenously injected with Sono Vue (2.4 mL; Bracco Imaging, Milan, Italy) and then flushed with 0.5 mL 0.9% saline. The regions of interest, including tumor nodules, ablation zones, and the surrounding parenchyma, were continuously observed for a minimal of 180 s after Sono Vue injection. MRI scans were performed on a 3.0‐T scanner (GE Healthcare, Waukesha, WI, USA). DCE‐T1‐weighted MRI was acquired after intravenous injection of Gd‐DTPA (Beilu Pharmaceutical Co. Ltd., Beijing, China). Post‐nsPEF imaging results were then reviewed by three certified radiologists to assess any tumor recurrence or complications. Overall survival (OS) was defined as the primary endpoint, and time to local recurrence within the ablated areas as the secondary endpoint. Primary point was patient death. Distant intrahepatic tumor recurrence was defined as the presence of a new tumor nodule outside the ablated areas.

### 2.4 Serum metabolomics analysis by liquid chromatography tandem mass spectrometry (LC–MS/MS)

Serum samples were thawed at room temperature. Then, 100 μL of each sample was added to a mixture of acetonitrile and methanol (v/v = 2/1) with 10 μL of internal standard consisted of 0.3 mg·mL^−1^ 2‐chloro‐l‐phenylalanine and 0.01 mg·mL^−1^ Lyso PC17:0, vortexed (10 s), ultrasonicated (10 min), and let stay at −20 °C for 30 min. Supernatant (300 μL) from each sample was dried in a centrifugal concentrator, redissolved in a mixture of methanol and water (v/v = 1/4), filtered, and then stored at −80 °C for metabolomic analysis. Quality control (QC) samples were prepared by pooling all of the samples in equal amounts.

Metabolomic analysis was carried out using a Nexera ultrahigh‐performance liquid chromatography (UPLC) system equipped with ACQUITY UPLC HSS T3 (Shimadzu, Kyoto, Japan), coupled to Q Exactive™ quadrupole orbitrap mass spectrometer (Orbitrap MS; Thermo Fisher Scientific, Waltham, MA, USA) equipped with heated electrospray ionization (ESI) source (Thermo Fisher Scientific, Waltham, MA, USA). The mobile phase consisted of solution A (0.1% formic acid in water) and solution B (0.1% formic acid in acetonitrile). The programmed gradient was set as follows: 0–0.01 min, 5% B; 0.01–2 min, 5% B; 2–4 min, 5–30% B; 4–8 min, 30–50% B; 8–10 min, 50–80% B; 10–14 min, 80–100% B; 14–15 min, 100–100% B; 15–15.1 min, 100–5% B; 15.1–16 min, 5% B. The injection volume was 2 μL and column temperature was set at 45 °C. The Q Exactive™ mass spectrometer was operated in both positive‐ and negative‐ion modes. Mass scan range was set at 125–1000 *m/z*. The resolution of full scan and HCD MS/MS scan was set at 70 000 and 17 500, respectively. The ion spray voltage was set at 3500 V in ESI^+^ and −3000 V in ESI^−^ mode. The sheath gas flow rate was set at 40 arbitrary units (a.u.) in ESI^+^ and 35 arb in ESI^−^ mode. Auxiliary gas flow rate was set at 10 a.u. in ESI^+^ and 8 a.u. in ESI^−^ mode. The capillary temperature was maintained at 320 °C. The QC samples were injected at regular intervals in the analytical sequence.

Pretreatment of the acquired raw data, including baseline filtration, peak identification and integration, retention time (RT) correction, peak alignment, and normalization, was performed using progenesis qi software (Waters Corporation, Milford, MA, USA). RT and exact molecular mass (*m/z*) were used to annotate metabolites based on the Human Metabolome Database (HMDB), Lipidmaps, METLIN database, and a customized database (Lu‐Ming Biotech Co. Ltd, Shanghai, China). The ESI^+^ and ESI^−^ data were merged, imported into an R package, and processed using an unsupervised orthogonal partial least square‐discriminant analysis (OPLS‐DA). Goodness‐of‐fit and the predictability of the OPLS‐DA model were assessed using 200‐time response permutation test and seven‐cross‐validation. It was validated that the OPLS‐DA model had a good predicative ability without overfitting (Fig. S1). Significant differential metabolites (SDMs) were defined as variables that were important for the projection (VIP) > 1.0 with a *P*‐value < 0.05. Kyoto Encyclopedia of Genes and Genomes (KEGG) database was used to perform functional annotation of SDMs.

### 2.5 Serum proteomic analysis by SWATH data‐independent acquisition

Proteins were extracted with depletion spin columns, quantified using a bicinchonininc acid (BCA) protein kit, and their qualities determined with SDS/PAGE electrophoresis. For enzymolysis, 50 μg protein of each serum sample were incubated with 3 μL sequencing‐grade trypsin (1 μg·μL^−1^; Hualishi Scientific, Beijing, China). The digested peptides were desalted by SOLA™ solid‐phase extraction (SPE) columns and lyophilized for examination of data‐independent acquisition (DIA).

Proteomic analysis was carried out on a 1100 UPLC system (Agilent, Santa Clara, CA, USA) coupled to a Q Exactive™ HF mass spectrometer (Thermo Fisher Scientific, Waltham, MA, USA). Reversed‐phase separation was achieved at a flow rate of 250 μL·min^−1^ with using dual mobile phases A (2% acetonitrile in water; PH = 10) and B (90% acetonitrile in water; PH = 10). The programmed gradient was set as follows: 0–10 min, 2% B; 10–10.01 min, 2–5% B; 10.01–37 min, 5–20% B; 4–8 min, 30–50% B; 8–10 min, 50–80% B; 10–14 min, 80–100% B; 14–15 min, 100–100% B; 37–48 min, 20–40% B; 48–48.01 min, 40–90% B; 48.01–58 min, 90% B; 58–58.01 min, 90–2% B; 58.01–63 min, 2% B. Isolated peptides were then lyophilized for further analysis.

Data‐dependent acquisition (DDA) and DIA analyses were operated on the Q Exactive™ HF mass spectrometer equipped with a Nanospray Flex source (Thermo Fisher Scientific, San Jose, CA, USA) connected via an EASY‐nLC 1200 interface system (Thermo Fisher Scientific, San Jose, CA, USA). Separation was conducted at a flow rate of 300 nL·min^−1^ using deal mobile phases: A (0.1% formic acid in water) and B (0.1% formic acid and 80% acetonitrile in water). The gradient was set as follows: 0 min, 5% B; 0–82 min, 37% B; 82–84 min, 90% B; 84–90 min, 90% B. The DDA MS scanning consisted of a full MS scan from 350 *m/z* to 1650 *m/z* with a resolution of 120 000 and AGC target of 3e6, and MS2 scans from 200 *m/z* to 2000 *m/z* with a resolution of 30 000 and AGC target of 2e5, moreover, for MS2, the isolation window was set at 1.4 *m/z* and the normalized collision energy was set at 27. For DIA experiment, the gradient was set as follows: 0 min, 5% B; 0–82 min, 44% B; 82–84 min, 90% B; 84–90 min, 90% B. Full DIA MS scan was set at 350–1250 *m/z* with a resolution of 120 000 and AGC target of 3e6, and for MS2 scans, the resolution was 30 000 and AGC target of 1e6, the isolation window was set at 26 *m/z* and the normalized collision energy was set at 28.

The raw data from DDA analysis were processed and aligned to the UniProt Homo sapiens database using Spectronaut Pulsar software (Biogenosys, Schlieren, Switzerland) to establish a reference library. Then, DIA raw data were matched with the DDA‐based library for protein quantification. For all results, the false discovery rate (FDR) was set at 0.01. Significant differential proteins (SDPs) were defined as those with a fold change (FC) > 1.2 and a *P*‐value < 0.05. Classification of the identified proteins was performed by the PANTHER (protein annotation through evolutionary relationship) classification system (www.pantherdb.org). Functions of the SDPs were annotated using KEGG database. Interactions between the SDPs were visualized with a protein–protein interaction (PPI) network analysis using a python package based on STRING (https://string‐db.org/) database. The same gene set used for gene set enrichment analysis (GSEA) further processed for gene ontology (GO) analyses.

### 2.6 Integrative analysis of metabolomics and proteomics

Top 100 SDPs and SDMs were ranked and selected based on *P*‐values. Correlations between the two sets were then computed using Pearson analysis, and the common biological functions shared by both sets annotated using KEGG analysis. The interaction network was constructed from KGML files.

### 2.7 C57BL/6 mouse model of hepatocellular carcinoma

Murine HCC line, Hepa1‐6 (RRIDs: CVCL_0327), was purchased from America Type Culture Collection (ATCC, Manassas, VA, USA). Hepa1‐6 were authenticated by the STR sequencing and verified as mycoplasma contamination free by ATCC. C57BL/6 mice at 6–8 weeks of age were obtained from Nanjing Biomedical Research Institute of Nanjing University. All mice were housed in the specified pathogen free (SPF) conditions with a 12‐h light/dark cycle. All mice had *ad libitum* access to food and water. All experiments were approved by the Ethics Committee at The First Affiliated Hospital School of Medicine, Zhejiang University School of Medicine (approval number: 2021‐360).

The orthotopic HCC model was established by injecting 10 μL of Hepa 1–6 cells (5 × 10^4^ cells·μL^−1^) suspended in a Matrigel/HBSS mixture (v/v = 50/50) into the left liver lobe of C57BL/6 mice. Ten days later, the inoculated mice with the largest diameter of HCC tumor < 10 mm were randomly assigned into nsPEF group (*n* = 30) and sham group (*n* = 20). Then the nsPEF ablation was performed as described in a previous report [7] using the following parameters: electric voltage, 10 kV, number of pulses, 200 pulses, pulse duration, 100 ns and frequency, 1 Hz. Mice in the sham group were subjected to a sham operation without nsPEF. Before sacrifice, samples were retrieved from HCC and adjacent tissues on day 3 and day 6 after the treatment for further analysis. Animal survival was recorded on the remaining mice until death occurred or sacrifice was necessary owing to tumor burden.

### 2.8 Single‐cell RNA sequencing analysis

Collected tissues were digested into single‐cell suspensions using a mouse liver/tumor dissociation kit (Miltenyi Biotec, Bergisch Gladbach, Germany) following manufacturer's instructions [16, 17, 18]. Single‐cell RNA sequencing was conducted on an Illumina platform and analysis was first performed as previously described [16, 17, 18, 19]. In brief, QC and filtering of raw data were performed (Fig. S2), and then principal component analysis (PCA) and graph‐based clustering analysis were conducted using the RunPCA and FindCluster in Seurat. Uniform Manifold Approximation and Projection for Dimension Reduction (UMPA) were performed to visualize the high‐dimensional data. Differentially expressed genes (DEGs) were defined as those with FC >1.5 and *P*‐value < 0.05. Pseudotime analysis was performed using r package monocle2. To explore the biological function differences of MNP subsets before and after nsPEF ablation, the gsva r package was used to investigate the biological signatures using MisgBD Reactome pathway. GSEA was performed to explore the biological signatures using MisgBD Reactome pathway. Additionally, the scmetabolism package was used to quantify the metabolic activity differences of MNP subsets. The cellphonedb was used to identify the ligand–receptor interactions between MNP subsets and T cell subsets, and the cell–cell interactome landscape was visualized using igraph and circlize r packages.

### 2.9 Mass cytometry analysis

Metal isotope‐tagged antibodies were prepared using an established protocol [16, 17], and their detailed information is listed in Table S1. The collected single‐cell suspensions were then analyzed using mass cytometry [16, 17] and visualized using flowjo software (version 10; Tree Star, Ashland, OR, USA).

### 2.10 Selectively depletion of liver Ly6C^+^ MNPs in mice

CCR2 antagonist (BMS CCR2 22; R&D Systems, Minneapolis, MN, USA) dissolved in absolute ethanol was administered at 100 μg per mice intraperitoneally, then this procedure was repeated at 24–48 h intervals to ensure continuous depletion. An equal volume of absolute ethanol was injected intraperitoneally as control treatment.

### 2.11 Statistics analysis

Diagnostic imaging results were processed and analyzed using dicom viewer (64 bit) software (Medixant Company, Poznan, Poland). graphpad prism (version 6.0; GraphPad Inc., La Jolla, CA, USA) and Statistical Package for the Social Sciences (spss) (version 19.0; IBM Corp., Armonk, NY, USA) were used for data analysis. Data are presented as means ± standard errors of the mean (SEM). *P*‐value < 0.05 indicated significant differences.

## 3 Results

### 3.1 Clinical characteristics of participants

**Fig. 1:**
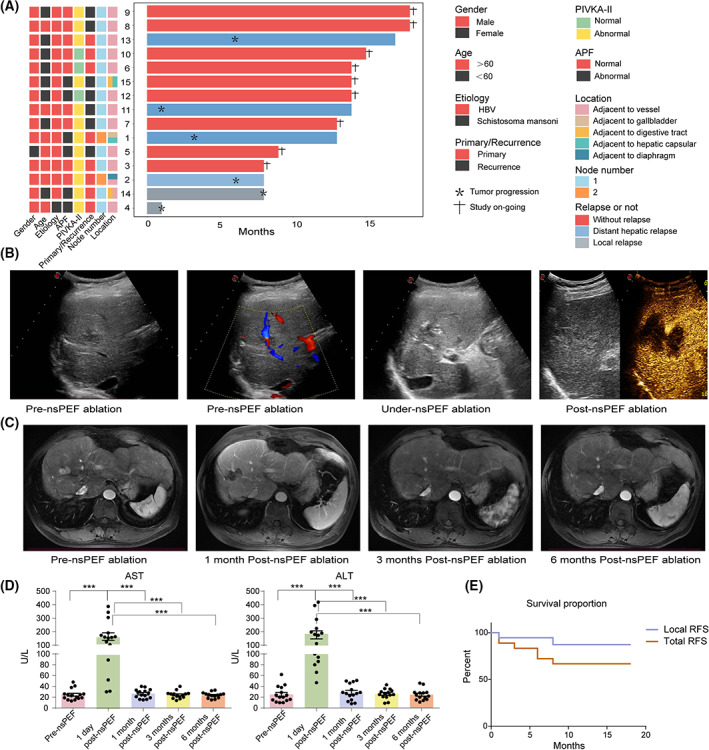
Safety and efficacy of nsPEF ablation for the treatment of liver carcinoma not amenable to thermal ablation. (A) Swimmer plot depicts the characteristics of enrolled patients. Each row represents a participant data. (B, C) Images in a 52 years old man with liver carcinoma located close to right hepatic veins. B‐mode US and CEUS images obtained pre‐, under‐ and post‐nsPEF ablation. Blue line indicates right hepatic vein and red line indicates portal vein (B). DCE‐T1‐weighted MRI obtained pre‐ and post‐nsPEF ablation (C). (D) Alterations of serum ALT and AST levels after nsPEF ablation (*n* = 15). (E) Graph shows the tumor recurrence free survival of 15 patients after nsPEF ablation. Data are means ± SEM from 15 repeats. The *P*‐value was determined by two‐tailed *t*‐test. ***P < 0.001.

Baseline characteristics of the 15 enrolled patients are summarized in Fig. 1A. The median age was 58 years (range 28–74). Fourteen of them had a history of hepatitis B virus (HBV) infection, and the remaining one (patient 4) had a history of schistosoma mansoni infection. Seven patients presented primary HCC, and the other eight patients had recurrent hepatic carcinoma. The median baseline alpha‐fetoprotein (AFP) and protein induced by vitamin K antagonist‐II (PIVKA‐II) levels were 9.8 ng·mL^−1^ and 74 mAU·mL^−1^ (range 1.1–735.5 ng·mL^−1^, 23–286 mAU·mL^−1^), respectively. The median size of the target nodules was 15 mm (rang 10–23 mm). A total of 17 liver nodules were ablated by nsPEF: 1 (5.9%) adjacent to gallbladder, 12 (70.5%) adjacent to major portal vein or major hepatic vein, 2 (11.8%) adjacent to major portal vein or both digestive tract and hepatic capsular, 1 (5.9%) adjacent to diaphragm, 1 (5.9%) adjacent to hepatic capsular.

### 3.2 Safety and efficacy of nsPEF ablation

For nsPEF ablations, two or three electrodes (median, two electrodes) were placed in parallel under US guidance (Fig. 1B). The median distance between electrodes was 16 mm (range, 11.3–18.8 mm). A total of 800–3200 pulses (median, 2000 pulses) were delivered. Repeat sessions, pull‐back or repositioning electrodes were applied as deemed appropriate by the operating surgeon. Detailed parameters of nsPEF procedures are summarized in Table 1.

At postsurgery checkups, gallbladder and gastrointestinal tracts surrounding the ablated tumors remained intact, and the veins within the ablation zone were well perfused (Fig. 1B,C). There was a transient increase of serum alanine transaminase (ALT) and aspartate aminotransferase (AST) at 1 day after nsPEF, indicating temporary impairment of liver function. Both ALT and AST declined to baseline levels by day 30 after the treatment (Fig. 1D). No other severe complications were observed, including sudden cardiac arrest, liver failure, myoglobinuria, bile leak, biliary obstruction, perforation, gastrointestinal tract bleed, or vein thrombus. Some patients experienced nausea, vomiting, mild abdominal pain, or fever, but none of them required medical interventions. All the patients recovered successfully before being discharged from the hospital.

The median follow‐up time was 14 months (range 1–18 months). Two (13.3%) of the15 patients developed local recurrence at 1 and 8 months after the treatment, respectively. Four (26.7%) patients presented distant recurrence after a median of 4.5 months (range 1–6 months). No extrahepatic tumor metastasis was observed. The 12‐month local recurrence‐free survival (RFS) was 86.7%, and the 12‐month overall RFS rate was 60% (Fig. 1E). These results demonstrate that nsPEF ablation is a safe and effective treatment option for liver cancer patients that are ineligible for thermal ablation. Importantly, nsPEF substantially reduced the risk of local tumor recurrence in this cohort of patients, compared to historical data of RFA, whose local tumor recurrence rate range from 48% to 58% [20, 21, 22], which indicated that the local efficacy of nsPEF ablation for HCC is encouraging.

Preclinical studies have shown that nsPEF could activate antitumor immunity [6, 7, 11], which corroborated with the findings in this study, However, the underlying mechanism remains to be elucidated. We started from examining the metabolomic and proteomic profiles of sera from patients that did not experience any tumor relapse up to 8 months after nsPEF.

### 3.3 Integrative metabolomic and proteomic analyses reveal that nsPEF ablation alters serum sphingolipid metabolism

**Fig. 2:**
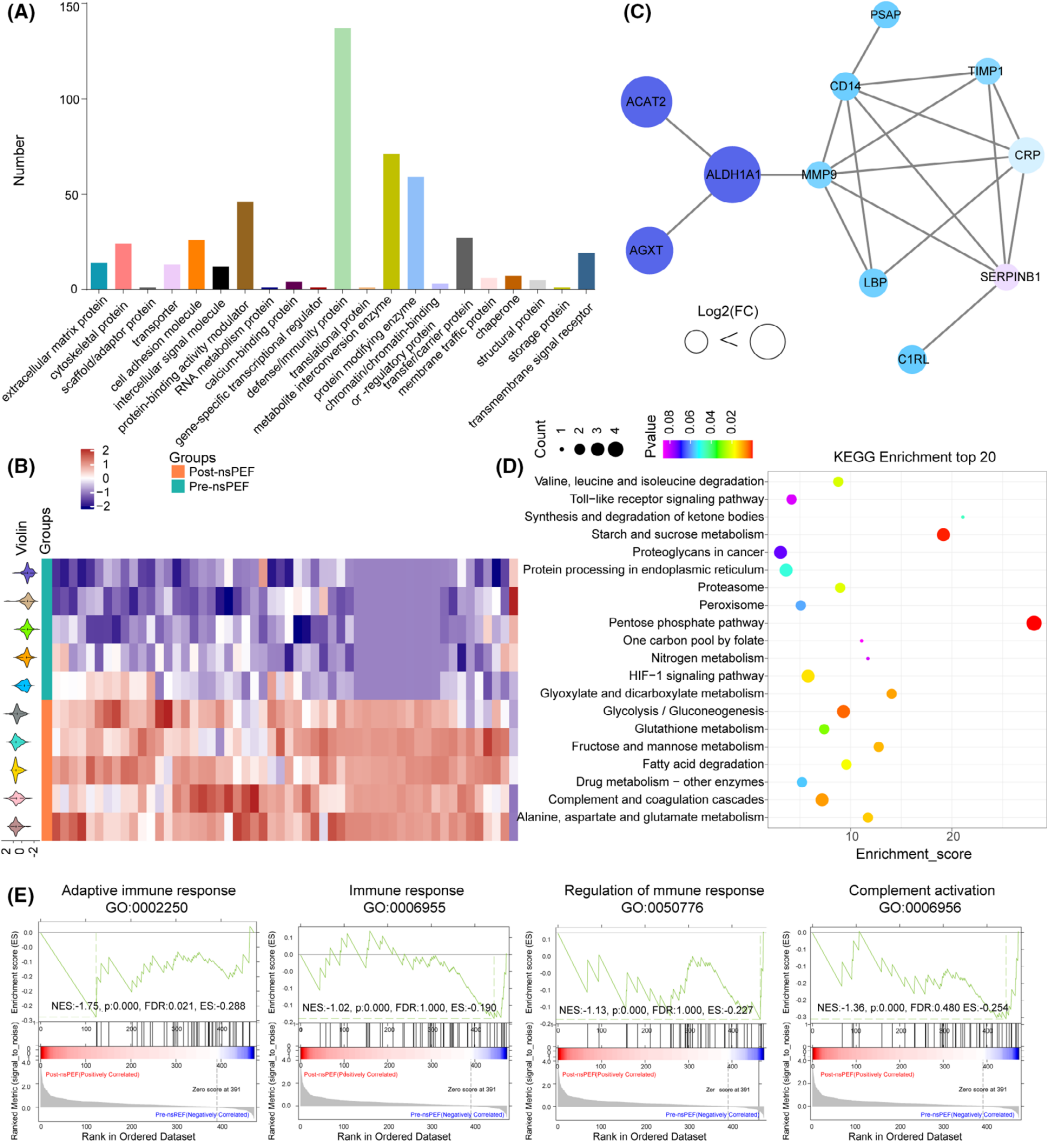
Depiction of serum proteomic alteration in liver carcinoma patients after nsPEF ablation. (A) Category of the identified proteins in the serum via the Panther classification system. (B) Heatmap showing the serum SDPs in serum pre‐ and post‐nsPEF ablation (C) PPI network analysis based on serum SDPs. (D) KEGG analysis of serum SDPs in liver carcinoma patients after nsPEF ablation. (E) GSEA analysis of serum SDPs after nsPEF ablation using GO gene sets.

In total, 542 proteins across 10 samples were analyzed, among which 478 proteins were annotated in the library, including: defense/immunity protein (25.3%), metabolite interconversion enzymes (13.1%), protein‐modifying enzymes (10.9%), and protein‐binding activity modulators (8.5%) (Fig. 2A). Fifty‐four proteins were significantly altered (FC 1.2‐fold and *P* < 0.05) by nsPEF, including one downregulated and 53 upregulated one (Fig. 2B; Table S2). SDPs related to lipid metabolism (e.g. prosaposin, acetyl‐CoA acetyltransferase, serine–pyruvate aminotransferase), and immunity (e.g. monocyte differentiation antigen CD14, complement C1r subcomponent‐like protein, lipopolysaccharide‐binding protein) were highly interconnected (Fig. 2C; Table S2). The top 20 enriched pathways in nsPEF‐treated patients are listed in Fig. 2D, with pentose phosphate pathway (PPP) as the top one. PPP regulates the formation and maintenance of CD8^+^ T memory cells [23] and modulates the polarization of macrophages [24, 25, 26, 27]. GSEA analyses showed that nsPEF significantly changed biological functions associated with immune response, regulation of immune response, adaptive immune response, and complement activation (Fig. 2E). Collectively, these results demonstrated that nsPEF modulated immune responses, and the resultant SDPs were mainly involved in immunity and lipid metabolism.

**Fig. 3:**
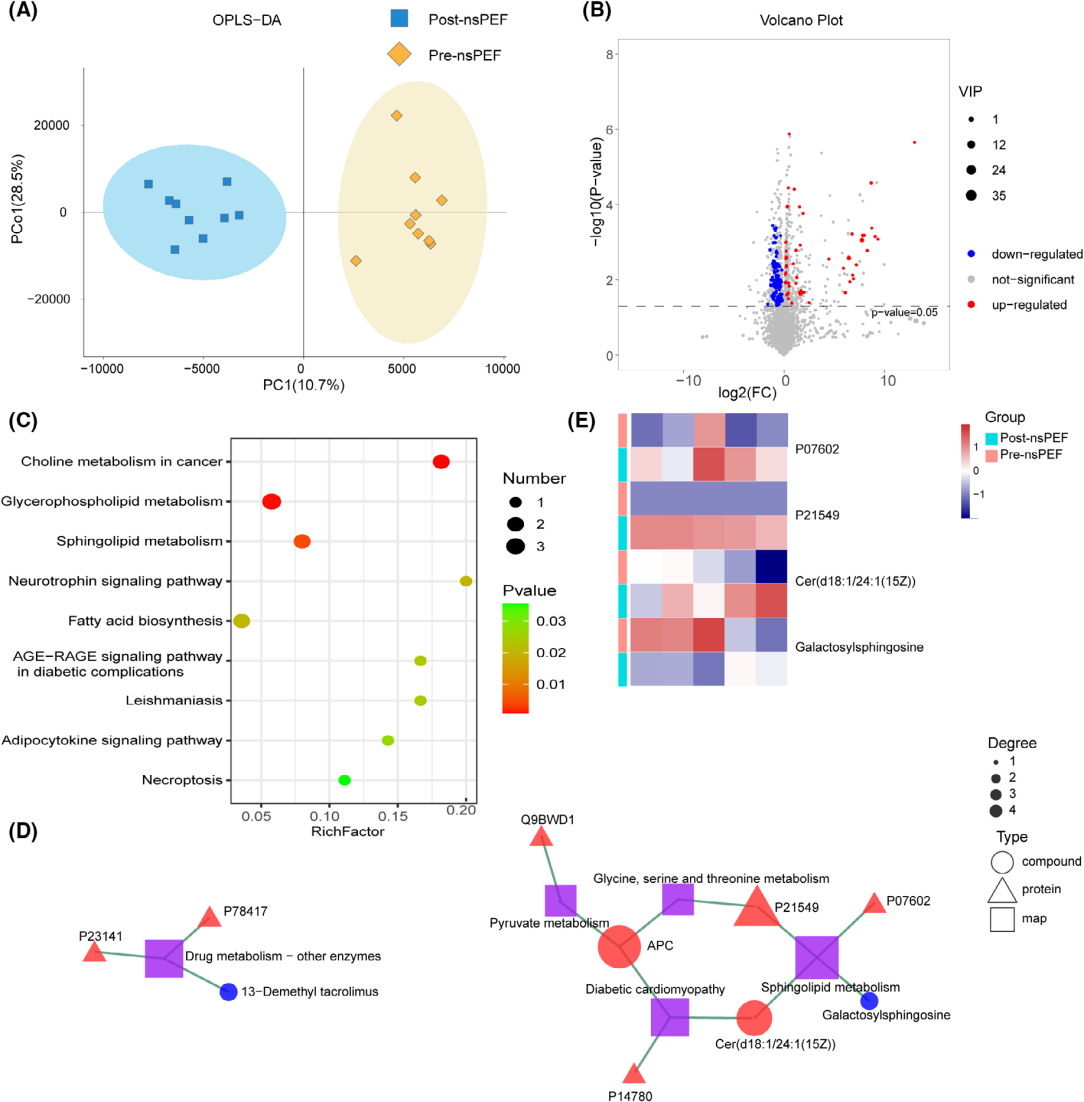
Serum metabolic alterations and key pathways revealed by the integrative analysis for liver carcinoma patients after nsPEF ablation. (A) Score plot of OPLS‐DA. (B) Volcanic map of SDMs in patients pre‐ and post‐nsPEF ablation. (C) KEGG analysis of serum SDMs in liver carcinoma patients after nsPEF ablation. (D) Integrative analysis based on serum SDPs and SDMs after nsPEF ablation. (E) Heatmap showing the key serum SDPs and SDMs involved in sphingolipid metabolism in five patients pre‐ and post‐nsPEF ablation.

Eighteen serum samples from nine patients pre‐ and post‐nsPEF were analyzed for metabolomic alterations. QC samples were first examined, which clustered closely in OPLS‐DA score plots and clustering map, validating that the data had good reproducibility and stability (Fig. 3A; Fig. S3).

In total, 5675 serum metabolites were identified, including 2407 negative electrospray ionization (ESI^−^) and 3268 positive ESI (ESI^+^) ones. As shown in Fig. 3B, 141 metabolites were significantly altered (variable importance in the projection (VIP) and *P* < 0.05) by nsPEF, including 83 downregulated and 58 upregulated (Fig. 3B; Table S3). KEGG analysis identified nine significantly enriched pathways, with choline metabolism in cancer, glycerophospholipid metabolism, and sphingolipid metabolism being the top three (Fig. 3C), all of which were related to lipid metabolism and involved in tumor development and recurrence [28, 29].

We then pooled the metabolomic and proteomic results and performed integrative pathway and network analysis. KEGG analyses identified five significantly enriched pathways (Fig. 3D), including sphingolipid metabolism, drug metabolism‐other enzymes, diabetic cardiomyopathy, pyruvate metabolism and glycine, serine and threonine metabolism. Sphingolipid metabolism was the most enriched pathway, including two upregulated SDPs (prosaposin and serine–pyruvate aminotransferase), one upregulated SDMs (Ceramide d18:1/24:1), and one downregulated SDMs (galactosylsphingosine) (Fig. 3E).

### 3.4 nsPEF ablation alters sphingolipid metabolism of immune cells (especially MNPs) rather than nonimmune cells

Metabolic alteration is a hallmark of HCC. Generally, the metabolic alterations of sera may reflect metabolic fluctuations of tissue. Increased evidence suggests that sphingolipid metabolism of various cells within tumor microenvironment (TME), including hepatocytes, liver sinusoidal endothelial cells (LSECs), cholangiocytes, hepatic stellate cells and immune cells, etc., is involved in the development and progression of HCC. Therefore, the next step is to explore which cell types within TME undergo sphingolipid metabolism alteration after nsPEF ablation.

**Fig. 4:**
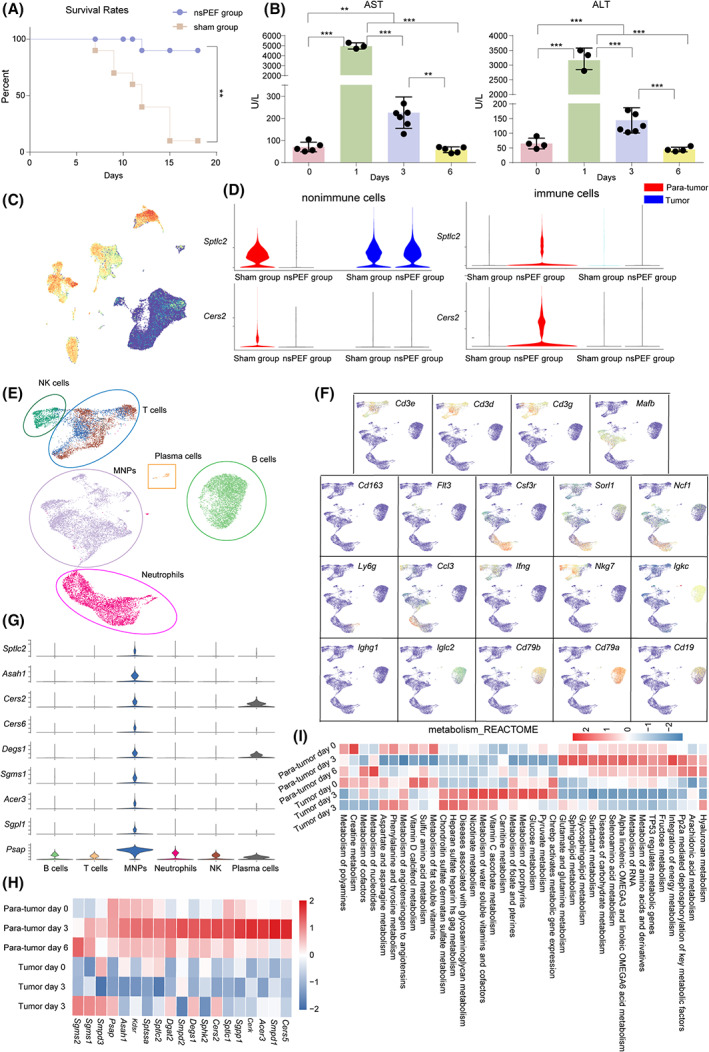
nsPEF ablation regulated the sphingolipid metabolism of mononuclear phagocyte system in mouse orthotopic HCC model. (A) Effects of nsPEF ablation on survival rate over time (sham group *n* = 10, nsPEF group *n* = 14). (B) Alterations of serum ALT and AST levels in mouse orthotopic HCC model after nsPEF ablation (day 0 *n* = 4, day 1 *n* = 3, day 3 *n* = 7, day 6 *n* = 4). (C) viSNE map showing the expression of *Ptprc* across all cells within TME of HCC. (D) Violin plots showing the expression of sphingolipids metabolism related genes in nonimmune and immune lineages. (E) viSNE map showing major immune lineages in mice HCC and Para‐ tumor tissues. (F) viSNE map showing the expression of marker genes across major immune lineages. (G) Violin plots showing the expression of sphingolipids metabolism related genes in major immune lineages. (H) Heatmap showing the sphingolipids metabolism related genes of MNPs in HCC and Para‐cancerous tissues at different time point. (I) The heatmap showing the significant metabolic pathway score of MNPs in HCC and Para‐ tumor tissues at different time points. Data are means ± SEM from at least three repeats. The *P*‐value in (A) was determined by Kaplan–Meier test, and in (B) were two‐tailed *t*‐test. **P < 0.01, ***P < 0.001.

We then strived to pinpoint the cell population that experienced a significant alteration in sphingolipid metabolism after nsPEF. Since liver biopsies are invasive and unfavorable for patients, we switched to murine orthotopic HCC models to this end. At day 18 post‐nsPEF ablation, the survival rate of nsPEF‐treated HCC‐bearing mice was significantly higher than that of the sham group (Fig. 4A). Similarly, the serum AST and ALT levels peaked at 1 day and then returned to the normal on day 6 posttreatment, (Fig. 4B), which was similar to liver enzyme alterations of liver carcinoma patients after nsPEF ablation.

We then profiled the paratumoral and intratumoral cells with scRNA‐seq and identified nonimmune and immune cells according to their expressions of *Ptprc* (Fig. 4C). After nsPEF ablation, nonimmune cells expressed significantly lower sphingolipids metabolism‐related genes in peritumoral tissues, while these genes expression were not significantly altered in tumor (Fig. 4D). And immune cells expressed significantly higher sphingolipids metabolism‐related genes in peritumoral tissues, while these genes almost not expressed in tumor (Fig. 4D). We then profiled the pritumoral and intratumoral immune cells with scRNA‐seq and identified the major immune lineages, including T cells, B cells, plasma cells, natural killer (NK) cells, neutrophils, and mononuclear phagocytes (MNPs) according to their expressions of marker genes (Fig. 4E,F). MNPs expressed significantly higher expression of sphingolipids metabolism related genes compared to other major lineages (Fig. 4G). The expressions of sphingolipids metabolism related genes were higher in peritumoral tissues than in tumor tissues, especially at day 3 post‐nsPEF (Fig. 4H). Metabolomic alterations visualized at single‐cell resolution also demonstrated that MNPs sphingolipid metabolism exhibited the highest activities in adjacent tissues at day 3 after nsPEF ablation (Fig. 4I; Table S4). Therefore, nsPEF ablation alters sphingolipid metabolism of MNPs, rather than the other immune cells and nonimmune cells.

### 3.5 nsPEF ablation‐induced sphingolipid metabolic alteration closely related to MNPs differentiation

**Fig. 5:**
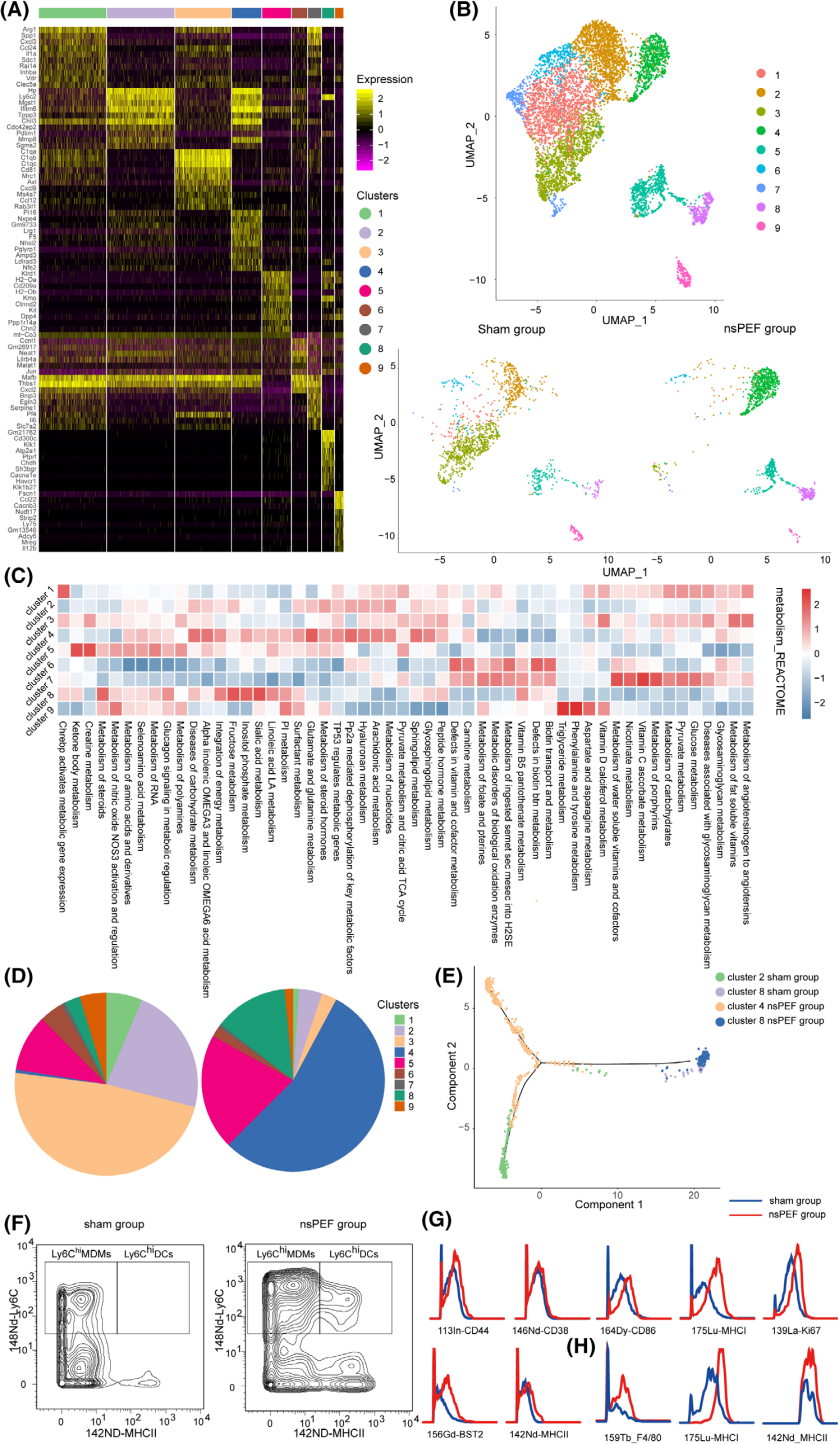
The influence of sphingolipid metabolism on the differentiation and function of MNPs. (A) Heatmap showing the top 10 signature gene expression of nine MNP clusters. (B) viSNE map showing the nine MNP clusters. (C) The heatmap showing the significant metabolic pathway score of nine MNP clusters. (D) Compositions of nine MNP clusters in mice Para‐tumor tissues pre‐ and 3 days post‐nsPEF ablation. (E) Pseudotime ordering of cluster 2, 4 and 8 in mice Para‐ tumor tissues over time. (F) Representative dot plots showing the percentages of Ly6C^hi^MDMs and Ly6C^hi^DCs in mice Para‐ tumor tissues. (G) Histograms of CD44, CD38, CD86, MHCI, Ki67, BST2 and MHCII expressions in Ly6C^hi^MDMs in mice Para‐ tumor tissues pre‐ and 3 days post‐nsPEF ablation. (H) Histograms of F4/80, MHCI and MHCII expressions in Ly6C^hi^DCs in mice Para‐ tumor tissues pre‐ and 3 days post‐nsPEF ablation.

Another unsupervised clustering of all MNP population identified a total of nine clusters (Fig. 5A). *Fcgr1* ^+^ monocytes/macrophages (including cluster 1–4) were categorized into *Ly6c2* ^−^ subsets (cluster 1 and cluster 3) and *Ly6c2* ^+^ subsets (cluster 2 and cluster 4). Additionally, the *Ly6c2* ^−^subsets had high expression of *C1q*, *Arg*, and *Mrc*, indicating that they were M1 macrophages [30]. On the other hand, the *Ly6c2* ^+^ subsets expressed high *Ly6c2* but low *Cd274*, while cluster 4 also expressed high level of *pglyrp1* that was could promote macrophage activation and phagocytosis [31]. Therefore, cluster 2 and cluster 4 represented Ly6C^hi^ inflammatory monocytes and monocyte‐derived macrophages (MDMs) [32, 33], respectively. Clusters 5, 8, and 9 had dendritic cell (DC) signatures with high expressions of *H2‐Oa*, *H2‐Ob*, and *Cd209a*. Cluster 8 also highly expressed *Ly6c2* (Fig. 5A,B; Table S5), suggesting that they were monocyte‐derived DC [32, 33].

Metabolic activities of the nine clusters at single‐cell resolution were shown in Fig. 5C. Interestingly, cluster 4 had the highest sphingolipid metabolic activity, followed by clusters 2, 3, and 8 sequentially (Fig. 5C; Fig. S4, Table S6). Clusters 2 and 3 were the main MNP populations in peritumoral tissues (22.6% and 47.9% of total cell population, respectively) prior to nsPEF (Fig. 5B,D). After nsPEF, the proportions of clusters 2 and 3 significantly decreased (4.1% and 2.9%, respectively), and those of clusters 4 and 8 significantly increased (from 0.6%, 2.8% to 54.7%, 13.0%, respectively). Therefore, the upregulation of sphingolipid metabolism post‐nsPEF might be closely associated with the increase in the proportions of clusters 4 and 8.

It was reported that that Ly6C^hi^monocytes were recruited into inflamed tissues and then differentiated into activated Ly6C^hi^MDMs and subsequently Ly6C^hi^DCs [17, 32]. Therefore, we surmised that cluster 2 (*Ly6c2* ^+^ monocytes) differentiated into cluster 4 (*Ly6c2* ^+^ MDM), which further transformed into cluster 8 (*Ly6c2* ^+^ DC). To validate this surmise, we depleted Ly6C^+^ MNPs by treatment with a CCR2 antagonist. Ly6C^+^ MDMs and Ly6C^+^ DCs were nearly absent at 3 and 6 days after the depletion of Ly6C^+^ MNPs, respectively (Fig. S5A,B). Indeed, all three clusters shared a similar metabolic signature (Fig. 5C), implying that they belonged to the same lineage. Pseudotime analysis also revealed a trajectory of *Ly6c*2^+^ MNP clusters that started from cluster 2, transposed into cluster 4, and ended at cluster 8 (Fig. 5E).

To further validate the scRNA‐seq data, mass cytometry analysis was performed on Ly6C^hi^MNPs (Fig. S6). In peritumoral tissues, there were less Ly6C^hi^DCs pre‐nsPEF, and more Ly6C^hi^MDMs and Ly6C^hi^DCs post‐nsPEF (Fig. 5F). The expressions of activated markers, CD86, CD44, CD38, and BST2, and major histocompatibility complex class I (MHCI) in Ly6C^hi^MDMs were all elevated after nsPEF (Fig. 5G), indicating the activation and antigen presentation of Ly6C^hi^MDMs. Ly6C^hi^DCs also expressed F4/80 (Fig. 5H), confirming their monocyte/macrophage lineage. Collectively, we have shown that nsPEF ablation activated *Ly6c2* ^+^ MDMs (cluster 4) and promoted their differentiation into *Ly6c2* ^+^ DCs (cluster 8).

### 3.6 *Ly6c2* ^+^ DCs promoted transformation of CD8^+^ T cells toward memory CD8^+^ T cells with potent antitumor effects

**Fig. 6:**
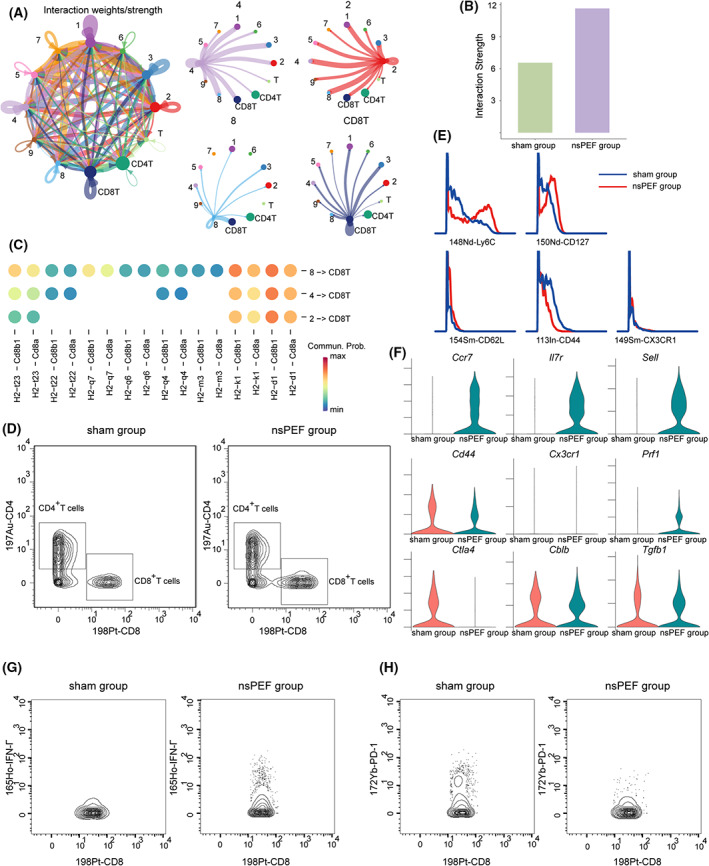
*Ly6c2* ^+^ DCs communicated with CD8^+^ T cells and promoted the formation of memory CD8^+^ T cells. (A) Cellular communication network of nine MNP clusters and T cell subsets. (B) Bar plot represents the interaction strength between *Ly6c2* ^+^ MNPs and CD8^+^ T cells. (C) Bubble heatmap indicating ligand‐receptor interactions between cluster 2, 4, 8 and T cell subsets. (D) Representative dot plots showing the percentages of CD4^+^ T cells and CD8^+^ T cells in mice Para‐cancerous tissues. (E) Histograms of Ly6C, CD44, CD62L, CD127 and CX3CR1 expressions in CD8^+^ T cells. (F) Violin plots showing the expression of *Ccr7*, *Il7r*, *Sell*, *Cd44*, *Cx3cr1*, *Prf1*, *Ctla4*, *Cblb*, *Tgfb1* in CD8^+^ T cell. (G) Representative dot plots showing the percentages of IFN‐γ^+^ CD8^+^ T cells in mice Para‐cancerous tissues. (H) Representative dot plots showing the percentages of PD‐1^+^ CD8^+^ T cells in mice Para‐cancerous tissues.

Given the ability of *Ly6c2* ^+^ MNPs subsets to prime antigen‐specific T cells immune response, we explored the communication among *Ly6c2* ^+^ MNPs subsets, CD8^+^ T cells, CD4^+^ T cells, and γδT cells according to the ligand‐receptor interactions (Fig. 6A–C). The interaction strength between *Ly6c2* ^+^ MNPs and CD8^+^ T cells was significantly higher in pre‐nsPEF than post‐nsPEF group (Fig. 6B,C). Therefore, we next characterized the distribution, phenotype, and functionality of CD8^+^ T cells in nsPEF ablation‐naïve HCC mice. Mass cytometry analysis revealed an increase in the proportions of CD8^+^ T cells after nsPEF (Fig. 6D), along with an upregulation of CD62L, CD127, Ly6C, and a downregulation of CD44 and CX3CR1 in the CD8^+^ T cells (Fig. 6E). A similar trend was observed in the gene expression by scRNA‐seq (Fig. 6F). Therefore, nsPEF reprogramed CD8^+^ T cells in naive adjacent tissues toward a memory CD8^+^ T cells [34, 35]. Interestingly, the proportions of memory CD8^+^ T cells were significantly reduced after selectively depletion of liver Ly6C^+^ MNPs in mice (Fig. S5C), which indicated that the *Ly6c2* ^+^ MNPs probably promoted transformation of CD8^+^ T cells toward memory CD8^+^ T cells.

Further, the expression of cytotoxic genes, *Prf1* (Fig. 6F), markedly increased after nsPEF ablation, and the expression of exhausted markers, *Ctla4* and *Cblb*, and immunosuppressive molecular *Tgfb1* were reduced (Fig. 6F). In line which these findings, mass cytometry analysis revealed an increase in the proportions of IFN‐γ^+^ CD8^+^ T cells (Fig. 6G), and a decrease in the proportions of PD‐1^+^ CD8^+^ T cells after nsPEF (Fig. 6H). Together, these data showed that nsPEF ablation promotes antitumor immune response by preventing exhaustion and maintaining the effector function of CD8^+^ T cells in peritumoral tissues.

In summary, we demonstrated that nsPEF ablation‐induced alteration of sphingolipid metabolism was closely correlated with the differentiation of *Ly6c2* ^+^ MNPs, and which involved in reprogramming of CD8^+^ T cells toward memory CD8^+^ T cells with potent antitumor effect in adjacent tissues at immune response in HCC.

## 4 Discussion

Thermal ablation exerts limited efficacy in patients with unresectable liver caners, for example, with tumor nodules close to gallbladder, gastrointestinal tracts, hepatic vessels, or portal vessels due to the “heat‐sink” effect, and often results in high rates of incomplete ablation or local recurrence [2]. nsPEF ablation is a novel treatment that works by nonthermal mechanism. We hereby, for the first time in clinical trials, shown that nsPEF spared the delicate peri‐tumoral structures, and substantially reduced the risk of incomplete ablation and local tumor recurrence in these patients as compared to the historical data of RFA [20, 21, 22]. Collectively, our results demonstrated that nsPEF could be a safe and effective alternative for the patients with unresectable liver cancer but ineligible to receive thermal ablation.

Although serum AFP and protein induced by PIVKA‐II are clinically approved biomarkers for monitoring the progression of liver cancer, their usefulness is hindered by low sensitivity [36, 37]. We examined the proteomic and metabolic alterations in patients' sera, and identified sphingolipid metabolism as the most significantly enriched pathway in those without tumor recurrence up to 8 months after nsPEF. Importantly, patients with significant changes in this pathway (defined as with a fold‐change of ceramide > 1.75 and a fold‐change of psychosine < 0.9) experienced a longer RFS (Fig. S7). Therefore, these two metabolites may have correlated with early recurrence in nsPEF‐treated liver cancer.

We went on to interrogate the mechanism underpinning the correlation between sphingolipid metabolism and nsPEF specific immune response. Sphingolipid are ubiquitously expressing molecules in eukaryotic membranes, maintaining cell integrity and orchestrating intercellular communications. Its metabolism was reported to regulate immunity in a variety of tumor types including liver cancer [38, 39]. Our data identified that ceramide and prosaposin are the central molecules involved in nsPEF‐induced sphingolipid metabolic alteration. Ceramide, a central metabolite of sphingolipid metabolism, is known to regulate immune responses in tumors [40, 41, 42]. Its expression in tumor‐associated macrophages (TAMs) mitigated ROS production by TAMs, repolarized them toward the antitumor M1 phenotype, and consequently enhanced antitumor immunity [41]. It also prevented the activation of MDSCs and enhanced the activity of CD8^+^ effector T cells [42]. Prosaposin, a precursor of sphingolipid activator proteins, is essential for the lysosomal degradation of sphingolipid and colocalizes with macrophages to mediate their inflammatory responses [43]. Collectively, our results suggested that sphingolipid metabolism in TME may have contributed to the regulation of immune response in liver cancer after nsPEF.

The activity of sphingolipid metabolism was further examined in the intratumoral cells, including immune and nonimmune cells, of nsPEF‐treated murine liver cancer. We found that nsPEF induced sphingolipid metabolic alterations in immune cells, especially MNPs, rather than nonimmune cells. It is also found to be closely related to the differentiation of *Ly6c2* ^+^ MNPs. The phagocytic and antigen‐presenting functions of MNPs are known to rely on sphingolipid metabolism [44, 45]. The accumulation of ceramide in the phagosomes of MNPs has been reported to promote phagosomal maturation [46]. Indeed, a large body of studies have shown that sphingolipid metabolism could drive the migration, activation, and polarization of monocytes/macrophages. Quintana et al. suggested that lactosylceramide facilitated the recruitment and activation of infiltrating monocytes, therefore responsible for the innate immune responses within the central nerves system [47]. In addition, sphingo‐1‐phosphate released from apoptotic tumor was a powerful chemoattractant for monocytes, and was involved in the polarization of macrophages toward the proinflammatory phenotype [48, 49, 50]. Therefore, sphingolipid metabolism maybe involved in the regulation of *Ly6c2* ^+^ MNPs differentiation through the above mechanisms; however, further investigations on the exact mechanisms are required.

## 5 Conclusions

In summary, we discovered that nsPEF ablation regulated the sphingolipid metabolism, which is closely related to the differentiation of *Ly6c2* ^+^ MNPs. High levels of *Ly6c2* ^+^ DCs promoted the formation of memory CD8^+^ T cells with potent antitumor effect.

## Conflict of interest

The authors declare no conflict of interest.

## Author contributions

TJ designed the experiment; JL performed the majority of experiments and analyzed the data and drafted the manuscripts; CF contributed to data visualization; XJ, ZS and LH collected clinical data and imaging; GT and JP performed the statistical analysis; XC conducted to the establishment of liver cancer mouse model. JZ and HC conducted clinical data and imaging analysis and language polishing. All authors read and approved the final manuscript.

### Peer review

The peer review history for this article is available at https://publons.com/publon/10.1002/1878‐0261.13372.

## Supporting information


**Fig. S1.** Permutation tests plots of OPLS‐DA model. OPLS‐DA: orthogonal partial least square‐discriminant analysis.

**Fig. S2.** Quality controls (QC) of scRNA‐seq data. (A) Number of unique molecular identifiers (UMI) per cell for each sample. (B) Genes number of single cells within each sample. (C) The percentage of mitochondrial genes in each sample. (D) Total genes number per cell in relation to UMIs are shown.

**Fig. S3.** Quality controls (QC) of LC‐MS/MS. (A) principal component analysis (PCA) score plot of QC samples. (B) Heatmap of QC samples.

**Fig. S4.** Sphingolipid metabolic activity of *Ly6c2* ^+^MNP subsets.

**Fig. S5.** Representative mass cytometry plots showing effects of Ly6C^+^MNPs depletion on Ly6C^+^MDMs (A), Ly6C^+^DCs (B), and CD8^+^memory T cells (C). MDMs: monocyte‐derived macrophages, DCs: dendritic cells.

**Fig. S6.** Gating strategy for Ly6C^+^MNPs.

**Fig. S7.** Alterations of sphingolipid metabolism predicted the outcome of HCC after nsPEF ablation. (A) Kaplan–Meier analysis for recurrence probability based on the FC of ceramide and psychosine. (B) Receiver operating characteristic (ROC) analysis of FC of ceramide and psychosine as predictive of recurrent status. AUC: area under the curve.

**Table S1.** List of metal isotope‐tagged antibodies for mass cytometry.
**Table S2.** SDPs in serum of liver carcinoma patients after nsPEF ablation.
**Table S3.** SDMs in serum of liver carcinoma patients after nsPEF ablation.
**Table S4.** Significant metabolic pathway score of MNPs in tumor and para‐tumor tissues at different time points.
**Table S5.** The expression of top 10 signature genes in nine MNP clusters.
**Table S6.** Significant metabolic pathway score of nine MNP clusters.

## Data Availability

All data generated or analyzed during this research are included within this article or from the corresponding authors upon reasonable request.
