# Supplementary figures and images for: Nanosecond pulsed electric field ablation‐induced modulation of sphingolipid metabolism is associated with Ly6c2 + mononuclear phagocyte differentiation in liver cancer
Source: Mol Oncol. 2023 Jan 21;17(6):1093–111. doi: 10.1002/1878-0261.13372 (PMC10257421; doi:10.1002/1878-0261.13372)

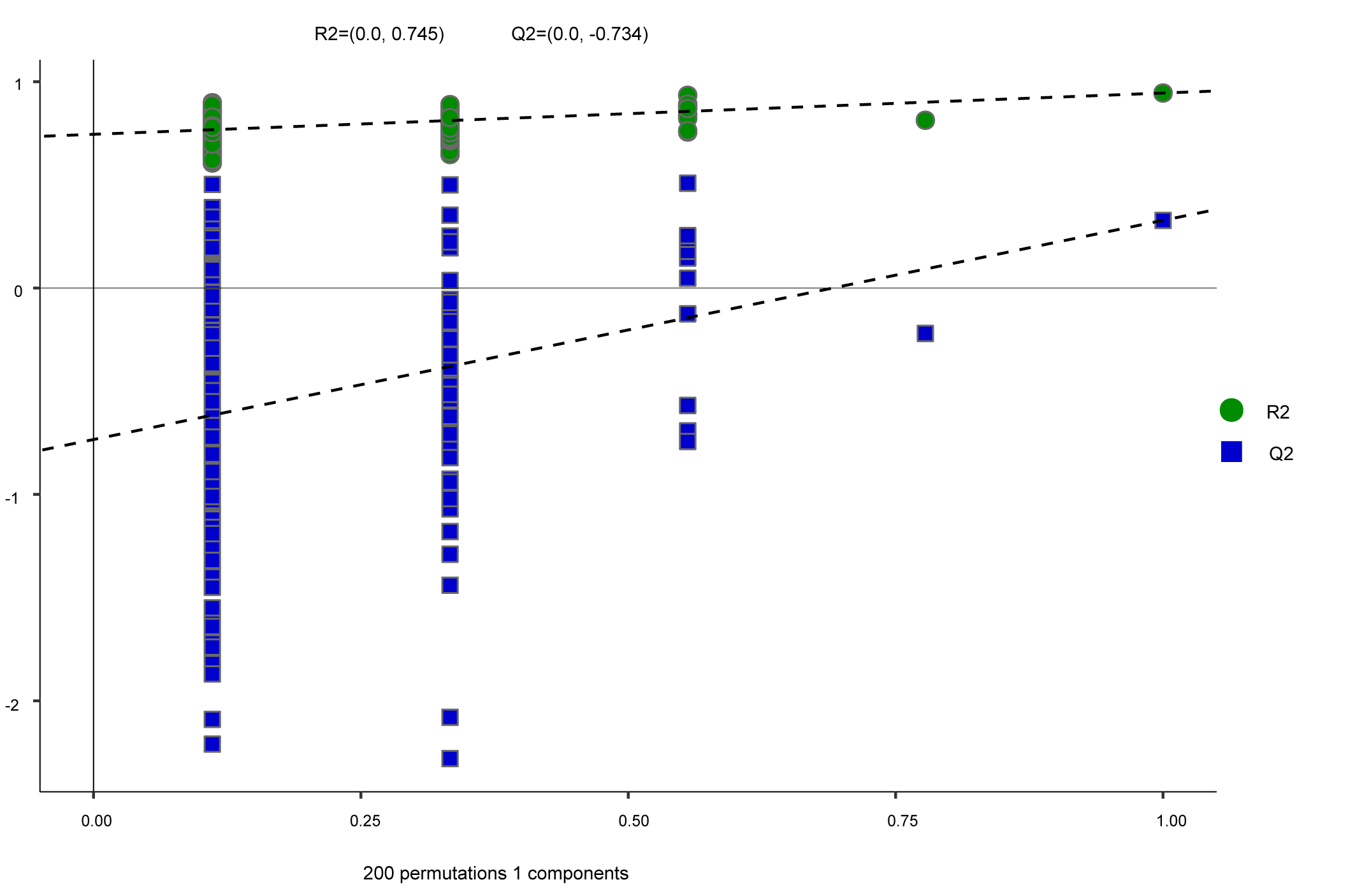

Supplement: Supplementary file 1 — Fig. S1. Permutation tests plots of OPLS‐DA model. OPLS‐DA: orthogonal partial least square‐discriminant analysis. [file MOL2-17-1093-s007.tif]

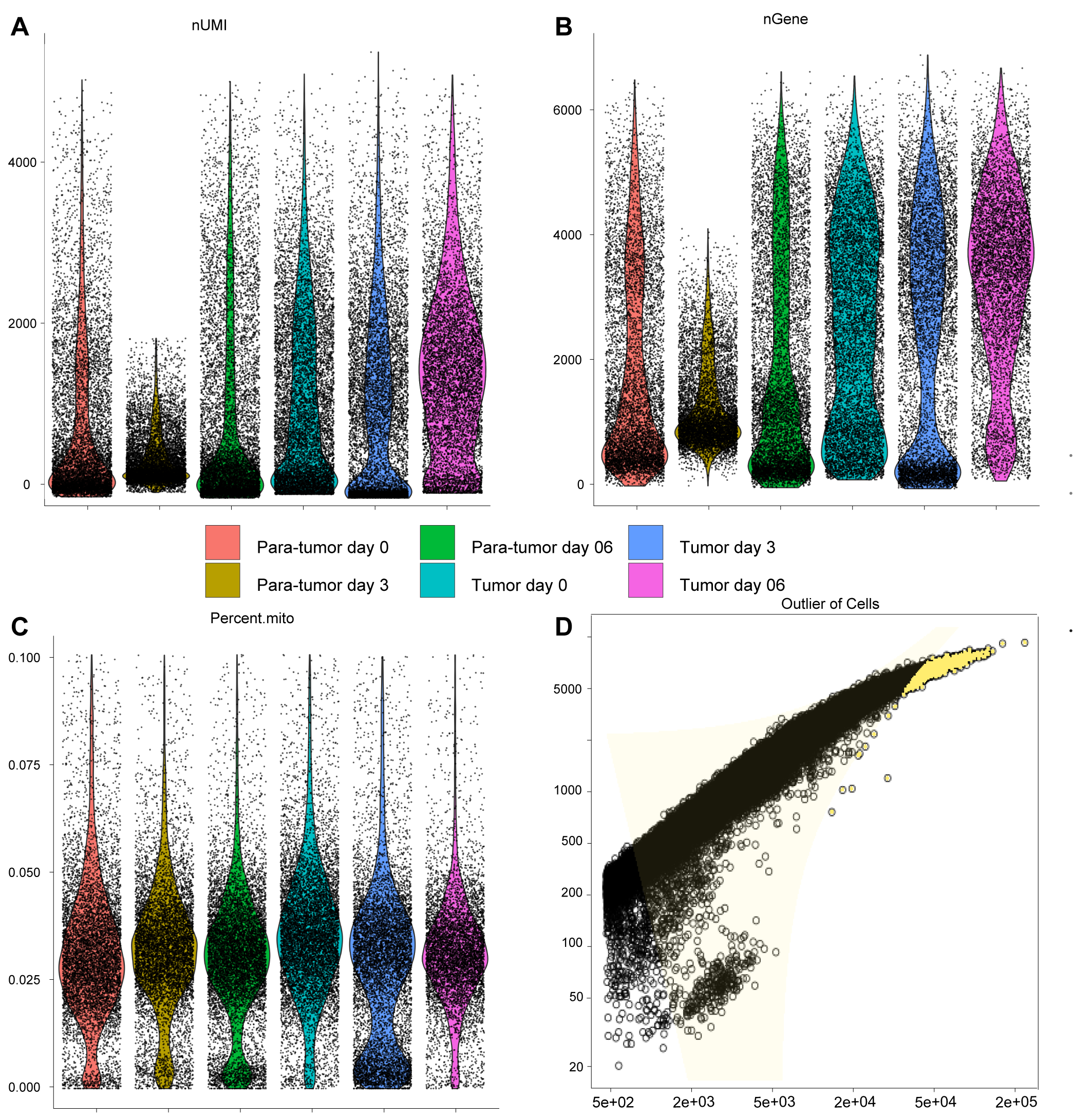

Supplement: Supplementary file 2 — Fig. S2. Quality controls (QC) of scRNA‐seq data. (A) Number of unique molecular identifiers (UMI) per cell for each sample. (B) Genes number of single cells within each sample. (C) The percentage of mitochondrial genes in each sample. (D) Total genes number per cell in relation to UMIs are shown. [file MOL2-17-1093-s001.tif]

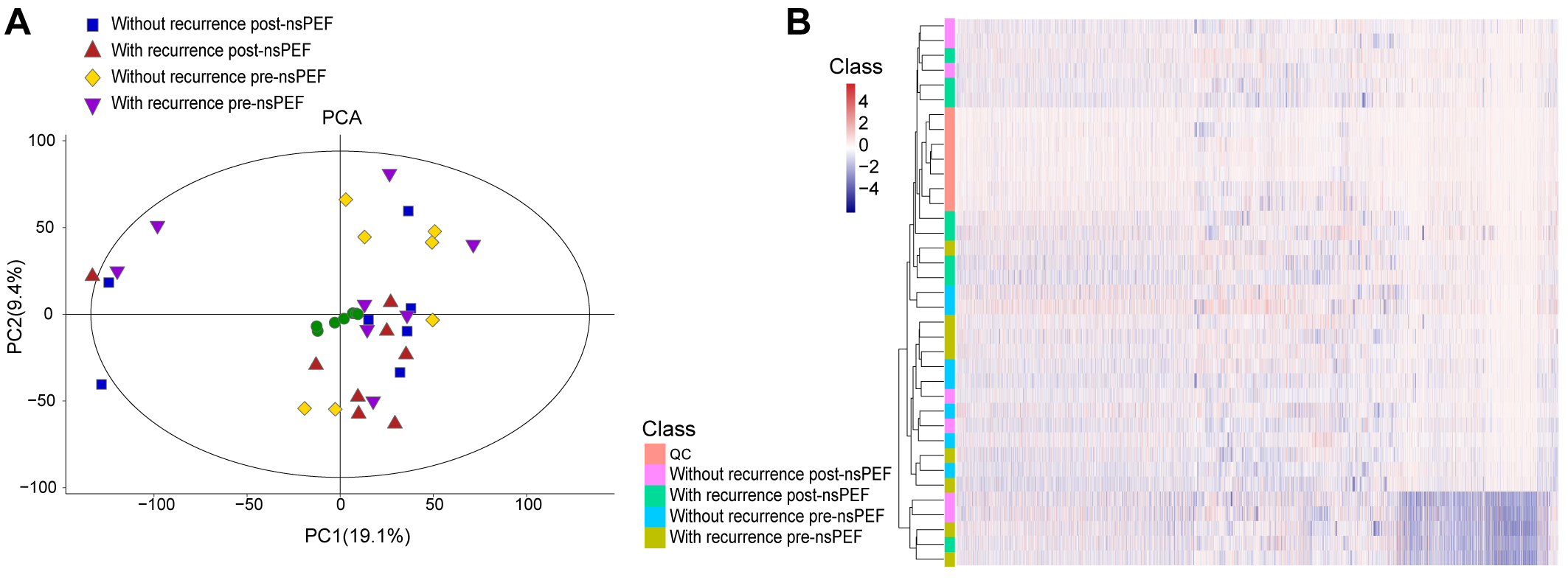

Supplement: Supplementary file 3 — Fig. S3. Quality controls (QC) of LC‐MS/MS. (A) principal component analysis (PCA) score plot of QC samples. (B) Heatmap of QC samples. [file MOL2-17-1093-s004.tif]

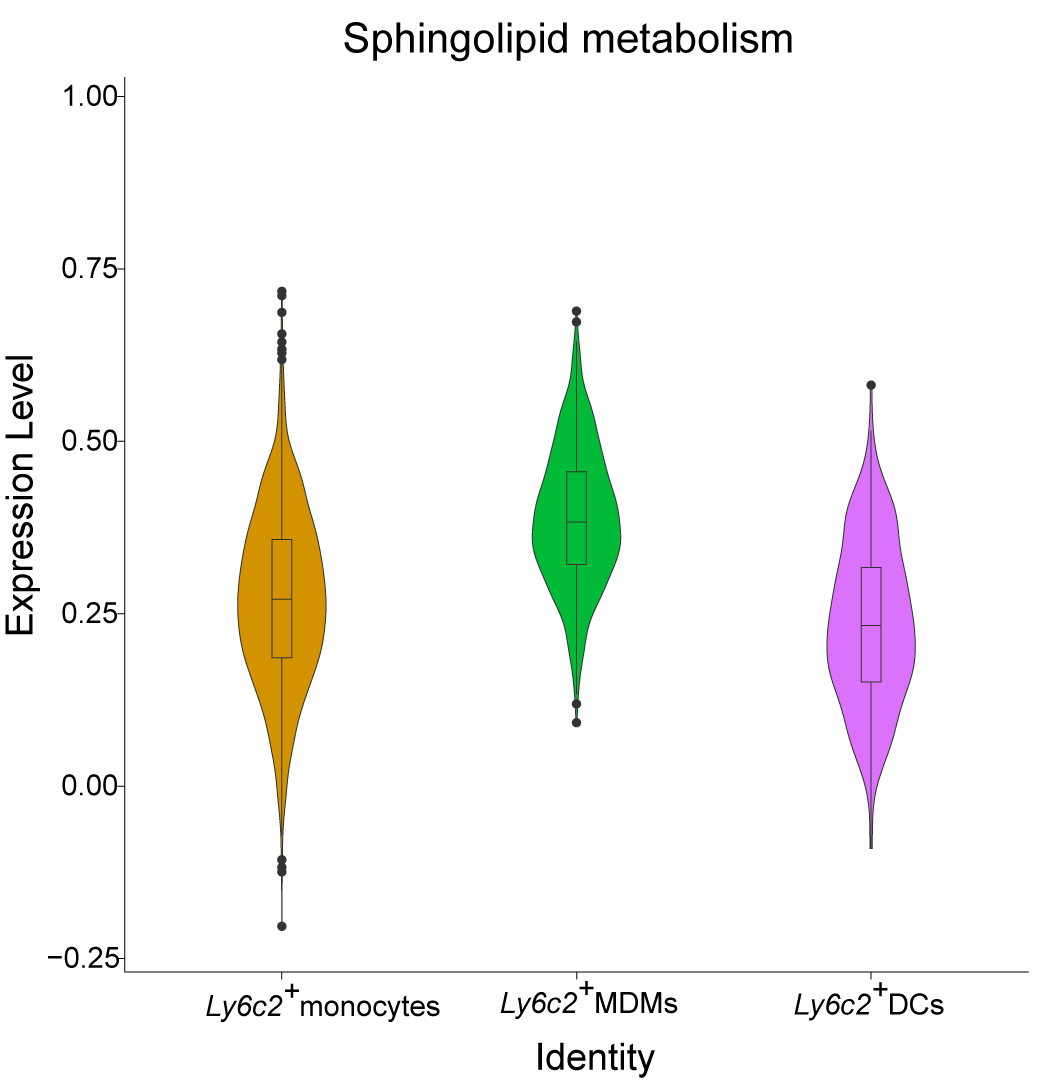

Supplement: Supplementary file 4 — Fig. S4. Sphingolipid metabolic activity of Ly6c2 +MNP subsets. [file MOL2-17-1093-s002.tif]

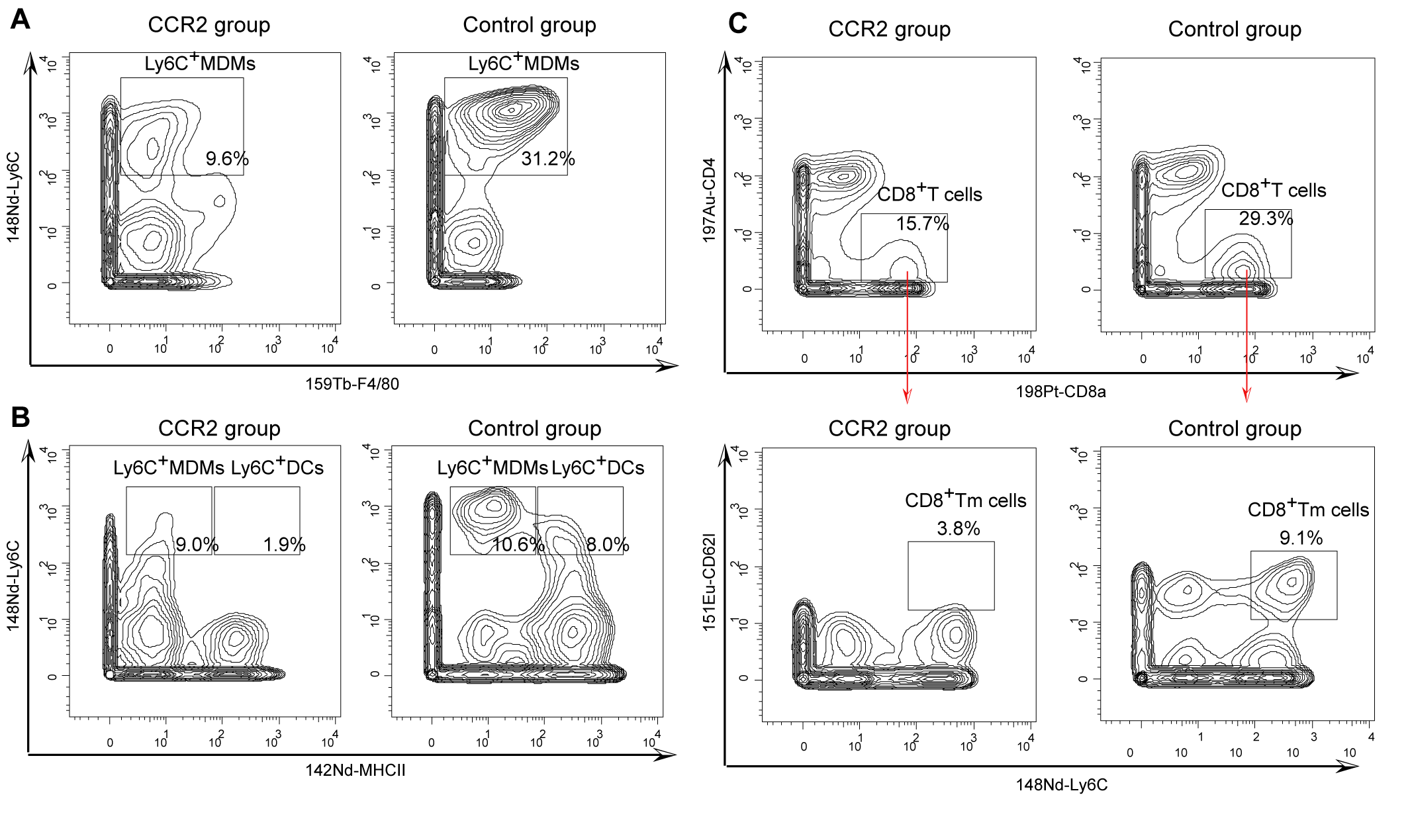

Supplement: Supplementary file 5 — Fig. S5. Representative mass cytometry plots showing effects of Ly6C+MNPs depletion on Ly6C+MDMs (A), Ly6C+DCs (B), and CD8+memory T cells (C). MDMs: monocyte‐derived macrophages, DCs: dendritic cells. [file MOL2-17-1093-s005.tif]

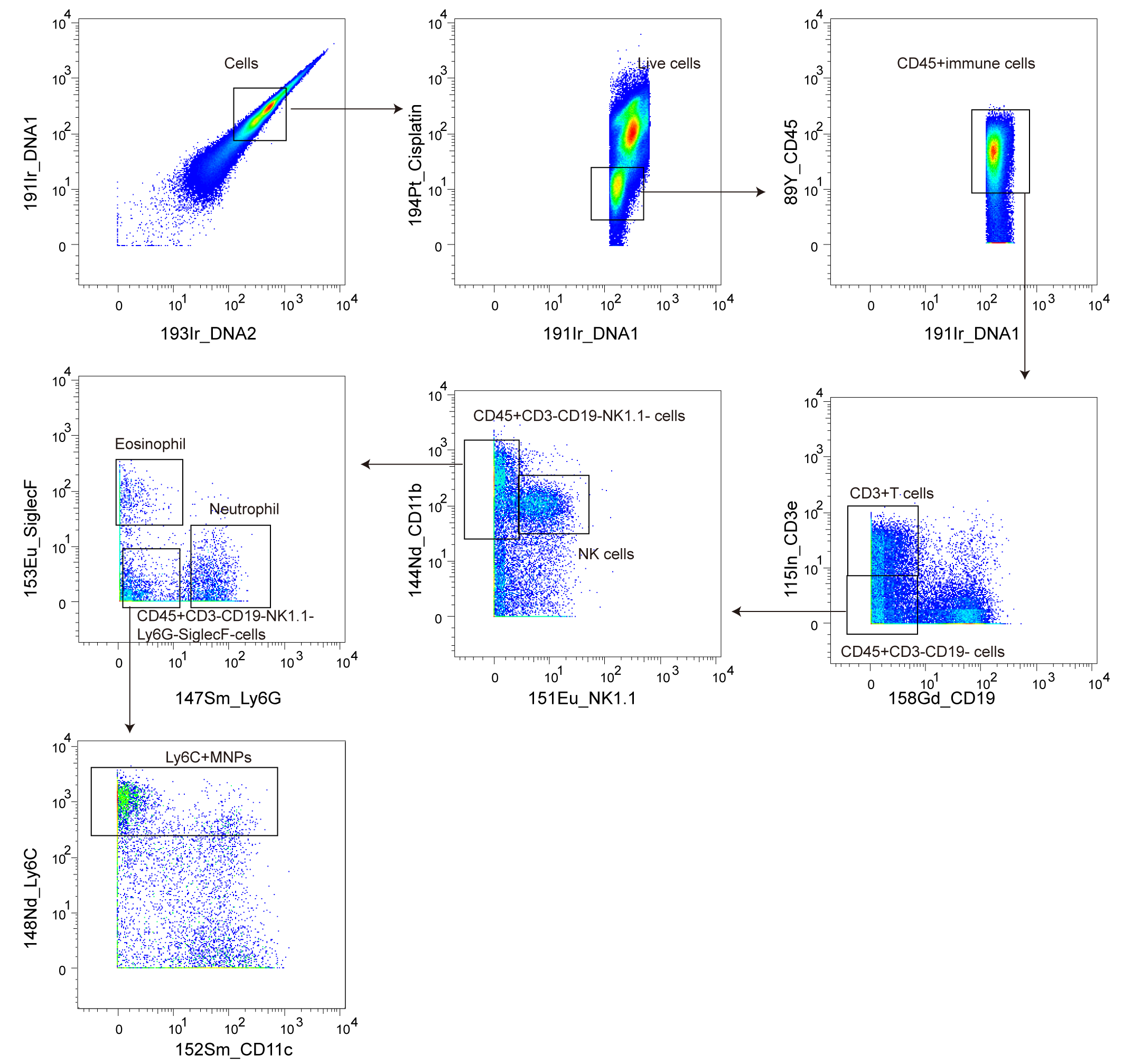

Supplement: Supplementary file 6 — Fig. S6. Gating strategy for Ly6C+MNPs. [file MOL2-17-1093-s006.tif]

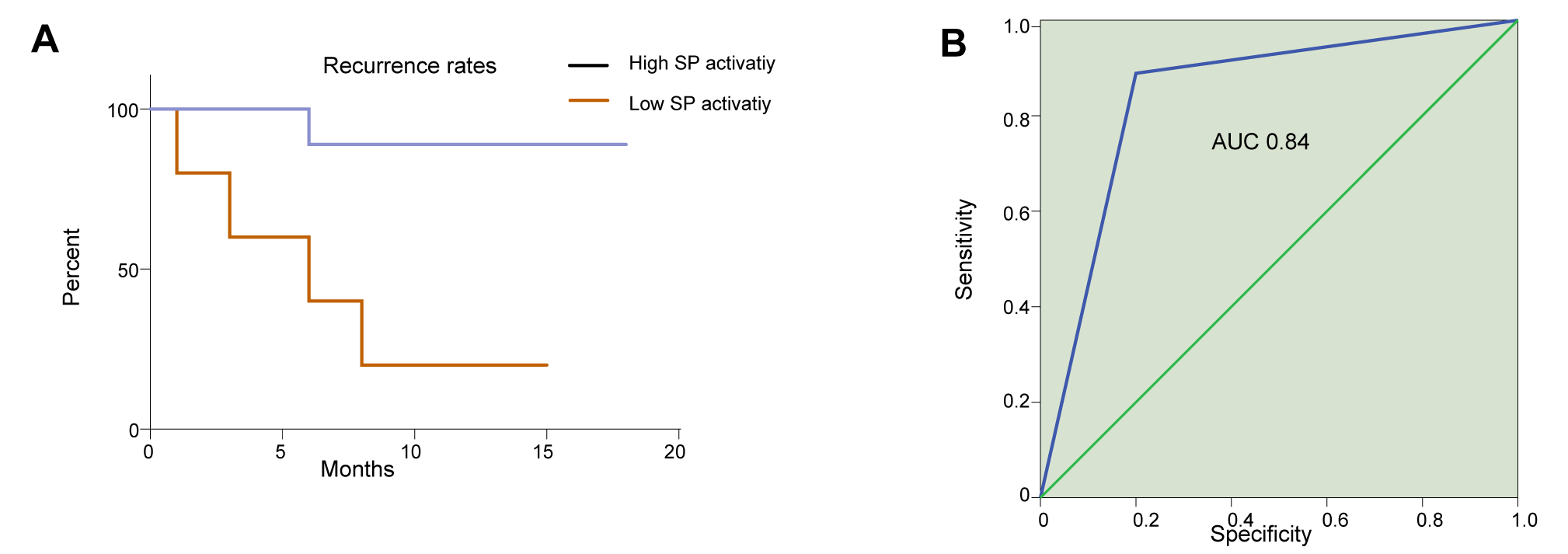

Supplement: Supplementary file 7 — Fig. S7. Alterations of sphingolipid metabolism predicted the outcome of HCC after nsPEF ablation. (A) Kaplan–Meier analysis for recurrence probability based on the FC of ceramide and psychosine. (B) Receiver operating characteristic (ROC) analysis of FC of ceramide and psychosine as predictive of recurrent status. AUC: area under the curve. [file MOL2-17-1093-s008.tif]
